# Rationalismus und Mystifikation: Zur formalen Pathetik des Dagegenseins

**DOI:** 10.1007/s41682-021-00095-9

**Published:** 2021-11-26

**Authors:** Robert Schäfer, Nadine Frei

**Affiliations:** Fachbereich Soziologie, Universität Basel, Petersgraben 27, 4051 Basel, Schweiz

**Keywords:** Kritik, Rationalismus, Conspirituality, Pathetik, Glaubwürdigkeitsproblem, Criticism, Rationalism, Conspirituality, Pathetics, Credibility problem

## Abstract

Der Beitrag stellt die Ergebnisse der Interpretation qualitativer Interviews mit Corona-Kritiker:innen dar. Gefragt wurde nach den Formen der Gesellschaftskritik, die sich in den Interviews manifestieren. Die Analysen zeigen (1), dass die Kritik auf einem rationalistischen Verständnis von Krisenlösung beruht. Die Tatsache, dass die Corona-Krise aus dieser Sicht nicht rational bearbeitet wird, wird als Indiz dafür gesehen, dass damit grundsätzlich etwas nicht stimmen kann. Auf dieses Problem reagiert die *conspirituality* der Kritiker:innen, eine Kombination verschwörungstheoretischer und esoterischer Vorstellungen, deren Einheit im Interesse am Geheimnisvollen gründet. Die Analysen erlauben es, (2) den spezifischen Stil der Maßnahmenkritik als formale Pathetik zu bestimmen: Substanziell bleibt sie relativ leer, wird aber umso leidenschaftlicher vorgetragen. Die rhetorischen Mittel sind dafür das Ziehen möglichst drastischer Vergleiche, die Romantik des heroischen Widerstandskampfs sowie der Anspruch, sich für das Wohl der Kinder zu engagieren. Schließlich wird (3) eine gesellschaftstheoretische Einbettung der Maßnahmenkritik angeboten, die mit Eisenstadt davon ausgeht, dass die moderne Gesellschaft von einer Erosion der Grundlagen aller Gewissheit geprägt ist, was zu einem grundsätzlichen Glaubwürdigkeitsproblem führt, das sich im Verlust des Vertrauens in zentrale gesellschaftliche Institutionen (Politik, Wissenschaft, Medizin, Medien) ausdrückt. In geradezu idealtypischer Weise kommt dies in der Maßnahmenkritik zum Ausdruck.

## Abstract (en)

The article presents the results of the interpretation of qualitative interviews with coronavirus critics. It focuses on the forms of social criticism manifested in the interviews. The analysis (1) shows that the critique is based on a rationalistic ideal of crisis resolution. The fact that the coronavirus crisis, from this point of view, is not dealt with rationally is seen as an indication that there is something fundamentally wrong with it. It is this problem that the *conspirituality* of the critics reacts to: a combination of conspiracy theory and esoteric ideas whose unity is based on the interest in the mysterious. The analysis (2) allows to determine the specific style of the critique of counter-measures as formal pathetics: Substantially, it remains relatively empty, while rhetorically it is emphasized all the more emphatically. The rhetorical means are the most drastic comparisons possible, the romanticism of the heroic resistance and the claim to be committed to the well-being of children. Finally, (3) we put forward a socio-theoretical embedding of the critique of counter-measures, which assumes with Eisenstadt that modern society is characterized by an erosion of the foundations of all certainty. This leads to a fundamental credibility problem, manifesting itself in the loss of confidence in central social institutions (politics, science, medicine, media). The criticism of counter-measures expresses that in an ideal-typical way.

## Einleitung

Die Proteste gegen die politischen Maßnahmen zur Verhinderung der Ausbreitung des Coronavirus haben von Anfang an die Frage aufgeworfen, wie die offensichtliche Heterogenität der Teilnehmer:innen zu erklären ist. Vor allem in Berlin (29.08.2020) und Leipzig (07.11.2020) waren auf den Demonstrationen Neonazis auffällig, und zahlreiche Personen, die der extremen Rechten zugeordnet werden können, wie zum Beispiel Attila Hildmann, Jürgen Elsässer oder Björn Höcke, unterstützen die Proteste lautstark. Auf der anderen Seite beteiligten sich daran viele Menschen aus dem anthroposophischen Milieu sowie Esoteriker:innen, die sich politisch eher links-grün verorten. Ein quantitativer Survey unter Mitgliedern von Telegram-Gruppen der Querdenken-Szene hat das vergangene Wahlverhalten und gegenwärtige Wahlabsichten abgefragt und gezeigt, dass die Proteste maßgeblich getragen werden von Personen, die von links kommen, nun aber nach rechts tendieren.^1^ Die Protestbewegung beschreibt sich selbst als Kollektiv, das weder links noch rechts stehe (vgl. Frei et al. 2021), sondern sich einfach für die Freiheit, die Selbstverantwortung, das kritische Hinterfragen etc. einsetze.^2^ So schildert etwa die 26-jährige Berufsschülerin aus unserem Sample ihre Erfahrung an Protestveranstaltungen: *„Der größte Teil von den Demo-Leuten ist ohne – wirklich mit Flagge oder Symbolen unterwegs gewesen, sondern als Mensch dort“*.^3^ Und der 33-jährige Logistiker, der sich selbst als Araber und Muslim bezeichnet, berichtet: *„Und wenn jetzt jemand anders eingestellt ist politisch als ich, dann ist das sein Ding. Der lässt mich in Ruhe, ich lasse ihn in Ruhe. Um das geht es ja nicht, es geht um die Sache. Und die Sache ist jetzt die Maßnahmen und ich meine, ich muss jetzt nicht mit allen Freunde sein, die dort teilnehmen, das ist mir schon bewusst und ja und mit allem einig sein mit diesen Leuten, die dort sind, aber ich finde jeder soll sein, wie er ist. Das Glauben, was er will, ja.“*^4^

Die soziologische Interpretation darf diese Selbstbeschreibungen natürlich nicht einfach naiv reproduzieren. Dennoch sollte sie sich auch nicht einfach darüber hinwegsetzen. Dieser Beitrag geht von der Beobachtung aus, dass es bei aller sozialstrukturellen und milieuspezifischen Diversität sowohl inhaltlich als auch stilistisch erstaunlich große Überschneidungen gibt. Was die Maßnahmenkritiker:innen eint, sind nicht ihre politischen Einstellungen,^5^ sondern (1) das, *was* sie sagen, und (2) *wie* es gesagt wird. Inhaltlich ist die Kritik strukturiert durch ein eigentümliches Spannungsverhältnis von Rationalismus und einer Haltung, die im Folgenden mit einem Ausdruck von Ward und Voas (2011) als *conspirituality* bezeichnet wird. Darunter ist die Komplementarität von verschwörungstheoretischen und esoterischen Überzeugungen zu verstehen.^6^ Die konspiritualistische Haltung enthält zahlreiche weltanschauliche Motive, die ihren ideengeschichtlichen Ursprung in der Romantik haben und sich als Kritik an der Moderne insbesondere gegen das wenden, was Weber „die Entzauberung der Welt“ genannt hat (vgl. Berlin 1999). Charakteristisch für die Kritik ist aber nicht nur ihr Inhalt, sondern auch ihr Stil. In unserer Studie verwenden wir dafür den Ausdruck *formale Pathetik*: Wenig Inhaltliches wird gesagt, das dafür aber leidenschaftlich.

## Methode und Sampling

Insgesamt wurden 16 Interviews geführt,^7^ die zwischen 60 und 90 min dauerten, sowie vier Kurzinterviews (ca. 40 min) auf Demonstrationen. Die Interviews wurden face-to-face oder per Zoom geführt, aufgenommen und wörtlich transkribiert. Sieben Interviews wurden in Gruppenarbeit zunächst sequenzanalytisch ausgewertet, um die grundlegenden Kategorien der weiteren Analyse rekonstruktiv zu bestimmen. Darauf aufbauend wurden die Transkripte mit MAXQDA codiert und die Kategorien durch systematische Fallkontrastierung zunehmend verfeinert. Es handelt sich insofern um ein Convenience Sampling, als die Studie darauf angewiesen war, dass sich Maßnahmenkritiker:innen für Interviews überhaupt zur Verfügung stellten. Sie wurden rekrutiert (1) durch Aufrufe in einschlägigen Telegram-Chatgruppen und auf Facebook, (2) durch Ansprechen und Flyerverteilung auf den ethnografisch untersuchten Demonstrationen in Konstanz und Leipzig bzw. auf einer kleineren Kundgebung in Basel und (3) durch direkten Mailkontakt, der auf Zuschriften nach Medienberichten über unsere Studie folgte.

Hier eine tabellarische Übersicht der Fälle, sortiert nach aufsteigendem Alter (Tab. 1):

**Table 1:** 

*Alter, Geschlecht*	*Beruf*	*Land*
24, w	Studentin Erziehungswissenschaften	D
26, w	Berufsschule, Eidgenössisches Fähigkeitszeugnis (EFZ)	CH
33, m	Logistik, Einkauf	CH
33, m	Student, Politiker	D
36, m	Schreiner, Politiker, Studium [Fach unbekannt]	D
36, w	Wissenschaftliche Mitarbeiterin (Soziologin, Dr. phil.)	CH
39, m	Videothekar, Blogger	CH
39, m	Wirtschaftsinformatiker	CH
44, m	Momentan ohne Anstellung, früher Sicherheitsbranche, Detailhandel, abgebrochenes Geschichtsstudium	CH
46, w	Psychologin	D
46, w	Diplom-Kauffrau (Personalleiterin in Pharma-Unternehmen) mit BWL-Studium	D
55, w	Früher: Unternehmerin, zuletzt: Seniorenbetreuungskraft	D
56, m	Ingenieur, Politiker	D
62, m	Gebäudeverwaltung	D (Wohnsitz DK)
62, w	Lehrerin	CH
72, w	Rentnerin, früher: Kaufmännische Angestellte, Weiterbildungen	CH
[Unbekannt], m	[Unbekannt: Kurzinterview auf Kundgebung Basel]	CH
[Unbekannt], m	[Unbekannt: Kurzinterview auf Demo Konstanz]	D
[Unbekannt], m	[Unbekannt: Kurzinterview auf Demo Konstanz]	D
53, w & [unbekannt], w	Kurzinterview auf Demo Konstanz, Erzieherin und [unbekannt]	D

Vorweg ist der empirische Befund zu erwähnen, dass es sich bei der Kritik an den aktuellen Corona-Maßnahmen um eine Kritik handelt, die nicht einfach auf eine bestimmte Sache zielt, sondern auch darauf abzielt, dass sie als Kritik verstanden wird bzw. so verstanden werden will. Das ist deshalb relevant, da sich in den Interviews gezeigt hat, dass die Tatsache, *dass* es sich um Kritik handelt, wichtiger ist als das, *was* kritisiert wird. Zudem ist vorwegzunehmen, dass ein Zweifel an der Existenz des Coronavirus nicht beobachtet werden konnte. Bestritten wird – z. B. durch die Behauptung einer Vergleichbarkeit mit einer normalen Grippe – dagegen die Gefährlichkeit des Virus sowie die Existenz einer Pandemie, was den Hintergrund abgibt für die Kritik an den politischen Maßnahmen, der wissenschaftlichen Forschung und der medialen Berichterstattung.

## Inhalt der Kritik

### Rationalistisches Modell von Krisenlösung

In allen Interviews findet sich die Überzeugung, dass nicht das Virus das Problem sei, sondern die Maßnahmen dagegen. Wie wird diese diskursive Problem-Verschiebung angestrebt? Das wichtigste Ergebnis unserer Analysen lautet, dass dafür ein rationalistisches Modell der Krisenbewältigung verwendet wird, an dessen Anspruch die Wirklichkeit notwendigerweise scheitern muss.^8^ Unter diesem Modell verstehen wir die Vorstellung, es gäbe strikt rationale Krisenlösungen. Ausgehend von Oevermanns Charisma-Konzept nehmen wir dagegen an, dass tatsächliche Krisen gerade dadurch gekennzeichnet sind, dass gängige Rationalitätsmaßstäbe außer Kraft gesetzt sind und deshalb in der Situation selbst noch gar nicht entschieden werden kann, ob sich die Bearbeitung der Krise einst als rational erweisen wird oder nicht (2016, S. 85ff.). Darin liegt die nicht stillzustellende Bewährungsdynamik als in sich dialektische Einheit von Entscheidungszwang und Begründungsverpflichtung. Diese Dynamik verweist auf den Zeitdruck, der akute Krisenbewältigungen immer charakterisiert: Es muss jetzt schon gehandelt werden, ohne dass diese Handlungen rational begründbar wären – allerdings mit grundsätzlichem Anspruch auf nachträgliche Begründbarkeit. In unserem Material lassen sich drei verschiedene Formen des Rationalismus unterscheiden. Aus der Perspektive der Befragten ist rational, (a.) was nicht logisch widersprüchlich ist, (b.) verhältnismäßig im Sinne einer Kosten-Nutzen-Rechnung ist und (c.) was in einem offenen Diskurs der Meinungsfreiheit zustande kommt. Im Folgenden wird dieses im empirischen Material vorgefundene rationalistische Modell der Krisenbewältigung ausgeführt und anhand ausgewählter Interviewsequenzen exemplifiziert.

- Formallogischer Rationalismus: Widerspruchsfreiheit

Auf die Einstiegsfrage, was sie an den Corona-Maßnahmen störe, antwortet die 62-jährige Lehrerin aus einer Schweizer Kleinstadt: *„In Bezug auf die Maßnahmen (…) das ist (…) also grundsätzlich sind diese Aussagen, die eben gemacht werden, die man gerade jetzt auch sieht, zur Weihnachtszeit, sie sind sehr widersprüchlich“.* Diese Form der Kritik wird in den unmittelbar folgenden Sätzen bestärkt. Sie moniert, dass den Maßnahmen ein *„logischer Faden“* fehle und man sie deshalb *„nicht wirklich zuordnen“* könne, was bei ihr eine *„Irritierung“* ausgelöst habe. Daraus lässt sich kehrseitig schließen, dass das Ideal der Krisenlösung, das hier unterstellt wird, nach dem Modell logischer Widerspruchslosigkeit und rationaler Schlüssigkeit konstruiert ist. Im Diskurs der Maßnahmenkritik besonders prominent ist auch die Kritik an der Widersprüchlichkeit wissenschaftlicher Aussagen bzw. deren politischer Umsetzung. Dass zu Beginn der Krise das Maskentragen als relativ nutzlos dargestellt wurde, später jedoch zu einem der wirksamsten Mittel gegen die Verbreitung des Virus und daher für obligatorisch erklärt wurde, wird als Widerspruch wahrgenommen, der darauf hinweist, dass mit der politischen Kommunikation grundsätzlich etwas nicht stimmen könne.^9^ So meint etwa die 72-jährige Rentnerin: *„Was ist denn da dahinter? Das ist für mich der Vorhang oder, wo ich sage he Moment, oder das ist doch nicht logisch, ist es denn logisch, dass dieses Virus auf ein Meter Zwanzig nicht da ist, aber wenn du aufstehst, ist das Virus- quasi überfällt dich, das ist einfach ein Witz oder ist einfach ein Witz“*. Analog dazu stört die 46-jährige Personalleiterin am meisten eine fehlende Konsistenz der verordneten Maßnahmen: *„Diese ständig wechselnden Parameter. Erst ist es das, als erstes ist es die Verdoppelungsrate, dann sind es die R‑Werte, dann sind es die Inzidenzen und all die Dinge. Es ist nicht konsistent in sich.“ *Diese Kritik an den Maßnahmen beruht auf dem rationalistischen Modell der Krisenlösung, das theoretische Kriterien ansetzt, um praktische Problemlösungsversuche zu beurteilen. Dass tatsächliche Krisen auch darin bestehen, dass aufgrund des Zeitdrucks formallogische Reflexionen über Stringenz und Konsistenz der Bewältigungsstrategien verunmöglicht werden, wird damit – ob strategisch oder nicht, kann nicht gesagt werden – ausgeblendet.

b. Kalkulatorischer Rationalismus: Verhältnismäßigkeit

Eine zweite Variante rationalistischer Kritik basiert auf dem formalen Kriterium der Verhältnismäßigkeit. Diese wird dabei gerade nicht als situatives Augenmaß in einer komplexen Krisensituation verstanden, sondern kalkulatorisch im Sinne einer simplen Kosten-Nutzen-Rechnung. So fragt etwa die 26-jährige Berufsschülerin rhetorisch:* „Schaden wir jetzt grad da einem ganz großen Teil von der Menschheit, um einen kleinen Teil der Menschheit zu schützen?“ *Der 44-jährige Arbeitslose, der sein Geschichtsstudium abgebrochen und sich autodidaktisch vielseitig informiert hat, konstatiert: *„Also die Verhältnismäßigkeit stimmt nicht und dann ist für mich ganz klar, es geht ja nicht um die Gesundheit, oder, haha (lacht), wenn die Verhältnismäßigkeit nicht stimmt, weil der Bundesrat ist nicht blöd, oder? All die Leute sind nicht blöd, die das organisieren, also ich meine, die sind alle sehr studiert und Fachleute und so und können Statistiken lesen und man weiß genau, es geht nicht um die Gesundheit, oder?“*^10^ Aus Sicht des Befragten sollte sich politisches Handeln durch wissenschaftliche Fundierung legitimieren lassen. Dass der Bundesrat und die *„Fachleute“* die Statistiken lesen können, heißt in diesem Kontext, dass das Verständnis der quantitativen Fakten zu verhältnismäßigem Handeln führen müsste – würde es denn tatsächlich um Gesundheit gehen. Dass Statistiken unterschiedlich interpretiert werden können und für die politischen Entscheidungsträger:innen nur eine mögliche Informationsquelle von vielen darstellen, ist in dieser Sichtweise nicht denkbar. Die praktische Logik der Politik – kollektiv bindende Entscheidungen zu fällen, gerade vor dem Hintergrund objektiv unentscheidbarer Auseinandersetzungen auf der Basis normativer Standpunkte – wird mit der theoretischen Logik der Wissenschaft gleichgesetzt, die auf dem Ideal der objektiven Entscheidbarkeit über Wahrheit und Falschheit beruht. Für die Berufsschülerin geht es darum, zur Beurteilung der Lage *„nicht nur eine nackte Zahl“* zu nehmen, sondern *„Zahlen in Relation“* zu setzen. Würde man nur richtig rechnen, so die implizite Annahme, ergäbe sich auch die richtige politische Maßnahme. Der Rationalismus, der sich dabei zeigt, ist nicht formallogischer Natur, sondern kalkulatorischer: Die adäquate Form der Krisenbewältigung, so ist dabei unterstellt, ließe sich berechnen. Dass tatsächliche Krisen darin bestehen, dass Kosten und Nutzen gerade nicht eindeutig berechnet werden können, da allgemein anerkannte Bewertungskriterien außer Kraft sind, wird dabei entweder nicht gesehen oder bewusst ignoriert.

c. Kommunikativer Rationalismus: Meinungsfreiheit

Ein verbreiteter Topos der Corona-Maßnahmen-Kritik ist die Betonung der Wichtigkeit der offenen Diskussion: *„Es ist einfach so, dass Menschen nicht mehr kommunizieren können, auch wenn sie ’ne andere Meinung haben, also das ist ja da auch das, dass ich sag’ mal meine Seite viel fordert. Dieser offene Diskurs kommt einfach nicht zu Stande und Menschen, die ’ne andere Meinung haben, werden sofort mit schlimmen Schimpfwörtern betitelt“. *In diesem Zitat ist auffällig, dass die Interviewte, eine 24-jährige Erziehungswissenschaftenstudentin, zwei Positionen gleichzeitig einnimmt. Sie ist Schiedsrichterin und Partei. Einerseits kann sie wie von oben beurteilen, dass diese Diskussion nicht zustande kommt, andererseits identifiziert sie sich deutlich mit ihrer *„Seite“* und verstärkt dadurch das Problem, das sie zu lösen fordert. Die Interviewte beklagt eine *„Spaltung der Gesellschaft“*, die von den Medien *„sehr geschürt“* werde*.* Darunter versteht sie, dass nicht *„regierungskonforme“* Meinungen *„unterdrückt“* und *„schlechtgemacht werden“*. Fraglos sind Meinungsfreiheit und Offenheit wesentliche Merkmale moderner Gesellschaften, allerdings sind sie immer auch beschränkt durch die Grenzen allgemein akzeptierter Rationalitätskriterien. Was die Maßnahmenkritik fordert, ist eine weite Ausdehnung, wenn nicht gar: Auflösung dieser Grenzen. Wirklich jede Meinung habe ihre Berechtigung und solle dementsprechend angehört werden. Wichtiger aber ist für die Analyse der Zusammenhang mit rationalistischen Vorstellungen. Hier tritt ein kommunikativer Rationalismus hervor, der auf einer Karikatur des Ideals eines herrschaftsfreien Diskurses beruht. Wie oben ausgeführt, zeichnen sich Krisensituationen dadurch aus, dass unter Zeitdruck entschieden werden muss und ein müßiges Abwägen erhobener Geltungsansprüche nicht möglich ist.

Jede Kritik, so unsere Ausgangslage, beruht auf der Identifikation eines Problems bzw. auf der erfolgreichen Suggestion eines solchen. Probleme bestehen immer dann, wenn eine Sache nicht so ist, wie es sein sollte, wenn sie also einem bestimmten Anspruch nicht genügt. Die Maßnahmenkritik unterstellt, nicht das Virus sei das Problem, sondern die Maßnahmen dagegen. Die Konstruktion dieses Problem wird rhetorisch erreicht, indem die Maßstäbe der Bewertung so gewählt werden, dass die tatsächlichen Maßnahmen der Krisenbewältigung zwingend davon abweichen müssen. Diese Form der Kritik beruht auf der Vorstellung, Krisen ließen sich rational bewältigen. Die Ideale, an denen die Befragten eine Krisenlösung messen, sind logische Stringenz, Verhältnismäßigkeit im Sinne einer statistisch korrekten Kosten-Nutzen-Kalkulation sowie ein herrschaftsfreies Abwägen aller möglichen Standpunkte. Da Krisen als solche indessen dadurch definiert sind, dass unter einem Zeitdruck gehandelt werden muss, der Widerspruchsfreiheit, genaues Durchrechnen und müßiges Diskutieren verunmöglicht oder zumindest mehr oder weniger stark erschwert, müssen die Maßnahmen, gemessen an diesen rationalistischen Maßstäben, notwendigerweise als inferior erscheinen.

Durch die Interpretationsarbeit ließ sich nicht abschließend klären, ob das durchgängig vorzufindende rationalistische Krisenlösungsverständnis von den Interviewpartner:innen tatsächlich vertreten wird oder ob es sich um eine rhetorische Figur handelt, um einen Strohmann, der zur Selbstinszenierung aufgebaut wird, um sich davon besser abgrenzen zu können. Letztlich ist das aber für unsere Analyse zweitrangig. In beiden Fällen dient die Explikation rationalistischer Vorstellungen wie ein Startblock für eine Haltung, die im Folgenden als eine Haltung beschrieben wird, die sich mit dem Konzept der *conspirituality* fassen lässt.

### Conspirituality

Durch das Messen einer Praxis an rationalistischen Maßstäben wird rhetorisch die Problematik der Maßnahmen konstruiert. Als Nächstes stellt sich die Frage, wie auf dieses Problem reagiert wird. Die hier vorgestellten Analysen zeigen dafür zwei verschiedene Varianten, die eng miteinander verbunden sind. Einerseits handelt es sich dabei um die (tendenziell pessimistische) Erklärung des gegenwärtigen Zustands, andererseits um die (optimistische) Explikation besserer Alternativen bzw. die Hoffnung auf eine künftige Erlösung. Für die Erklärung werden in den Interviews verschwörungstheoretische Deutungen entfaltet, die Alternativen beruhen auf esoterischen bzw. anthroposophischen Ideen. Eine Möglichkeit, die Komplementarität dieser beiden Deutungsmuster mit einem Begriff zu fassen, ist das Konzept der *conspirituality* (Ward und Voas 2011; Asprem und Dyrendal 2015; Schließler et al. 2020). Darunter wird die Einheit der beiden Überzeugungen verstanden, dass (a) eine geheime Gruppe die politische Ordnung kontrolliert und (b) sich die Menschheit vor einem unmittelbar bevorstehenden Paradigmenwandel befindet, der darauf beruht, dass immer mehr Menschen spirituell erwachen und so fundamentale Bewusstseinsveränderungen erleben: „the inner self must change before the outer world can“ (Ward und Voas 2011, S. 112).^11^ Die beiden Elemente der *conspirituality* werden hier zunächst gesondert dargestellt, um anschließend die Frage zu beantworten, was u. E. ihre Einheit ausmacht.

- Verschwörungsdenken

Der Übergang vom rationalistischen Modell von Krisenlösung zum Verschwörungsdenken verläuft so fließend, dass der Eindruck entsteht, jenes sei geradezu eine Bedingung für diese. Jedenfalls funktioniert das rationalistische Krisenbewältigungsmodell als Hintergrund, vor dem allfällige Widersprüchlichkeiten überhaupt als Indizien für etwas anderes gesehen werden können, in diesem Fall für eine unterstellte Verschwörung (Verschleierung, Lüge, Irreführung etc.). So führt die Lehrerin, die ihre Irritation aufgrund der logischen Widersprüchlichkeiten der Maßnahmen betont, ihre Ausführungen fort: *„Da frage ich mich halt, warum ist das nicht logisch (…) wenn ich das jetzt, wenn ich das jetzt schaue, wo dass man zum Beispiel nicht logische Äußerungen macht, zu welchen Zeiten oder in welchen Ländern, dann hat das eben meistens mit etwas zu tun das, das man vertuschen will zum Beispiel oder man führt etwas im Schild und das gibt einem dann so dieses Gefühl, stimmt etwas nicht?“* Fehlende Logik interpretiert sie generell als Zeichen für Verschleierungsabsichten.^12^

Alle Verschwörungstheorien gehen davon aus, dass es hinter der offiziell dargestellten Wirklichkeit eine andere Wirklichkeit gibt, die den normalen Leuten verborgen ist und zu deren Aufdeckung sie selbst beitragen: Nichts ist, wie es scheint.^13^ Diese andere Wirklichkeit ist typischerweise ein mehr oder weniger enger Zirkel, der mit der Verschleierung der tatsächlichen Zusammenhänge sinistre Absichten verfolgt. Prominente Kandidaten für die Zuschreibung solcher Absichten sind Juden, Jesuiten, Freimaurer, Illuminaten, die „Bilderberger“, aber auch Geheimdienste und Regierungen („Deep State“).^14^ Im Zusammenhang mit der Covid-Pandemie spielen Bill Gates, die WHO, die Pharmaindustrie sowie das World Economic Forum bzw. Klaus Schwab eine zentrale Rolle.^15^ In unseren Interviews hat sich allerdings gezeigt, dass es auf diese Akteure gar nicht so sehr ankommt. Besonders deutlich manifestiert sich diese Haltung bei der 46-jährigen Personalleiterin. Überzeugt davon, dass bei der ganzen *„Angstmacherei“ *etwas *„dahintersteht“*, und im Bewusstsein, dass sie dadurch den Weg ins Verschwörungsdenken einschlägt, hält sie unmissverständlich fest: *„Es ist mir schnuppe, ob da ein Klaus Schwab da ’ne Weltregierung oder nicht haben will, ist mir egal. Den Menschen muss am Ende gut gehen“.* Es ist also nicht wichtig, wer genau die Verschwörer sind und wer diese Krise inszeniert hat. Häufig bleiben die Beschreibungen der angenommenen Verschwörung erstaunlich dünn und reduzieren sich auf die allgemeine Annahme, wie es der 62-jährige Gebäudeverwalter ausdrückt, *„mächtiger Gruppen im Hintergrund“*. Daher ist wichtiger als die Frage, *wer* eigentlich dahintersteckt, die Behauptung, *dass* jemand dahintersteckt. Wichtig ist, dass nichts ohne Grund passiert, sekundär ist hingegen, was genau dieser Grund ist.^16^ Exemplarisch dafür ist folgende Sequenz aus dem Interview mit dem 56-jährigen Ingenieur, der auf die Frage nach den Gründen seiner Annahme einer systematischen medialen Desinformation antwortet: *„Also warum das geschieht, da müssen Sie mich nicht fragen, na, da müssen Sie die Medien fragen. Na, ich weiß ja nicht, was die Beweggründe von denen ist. Ich weiß nur, dass es passiert“.* Die Erziehungswissenschaftlerin meint: *„Also ja klar, natürlich hab’ ich dazu sehr, sehr viele Theorien dazu und hör’ auch sehr viele Theorien, aber das ist was (..) ich kann dazu keine hundertprozentige Antwort geben, weil ich sie selbst für mich noch nicht gefunden hab’.“* Und die Rentnerin formuliert: *„Oder wer hat da ein Interesse, das sind wieder die Fragen, die hinter dem Vorhang, aber dieser Vorhang ist noch zu dick für mich“.* Es wird durch dieses Verschwörungsdenken weniger ein Geheimnis gelüftet, sondern kommuniziert, dass es ein Geheimnis gibt.^17^ Für die Analyse von Verschwörungsdenken stellt sich hier Frage, worin die Attraktivität solcher Behauptungen gründet. Aus religionssoziologischer Perspektive ist zu vermuten, dass die Vorstellung, dass die weltlichen Ereignisse einem größeren Plan folgen, es erlaubt, den Verlauf der Weltgeschichte der Zufälligkeit und Kontingenz zu entheben, wodurch ihr auch ein tieferer Sinn zukommt.^18^ Was immer dieser sein mag, immerhin gibt es einen Sinn. Aus dieser Sicht erscheint das Verschwörungsdenken weniger als Kritik denn als Rechtfertigungsmuster, als eine Form der Kosmodizee (vgl. Schäfer 2021). Die Funktion der Kosmodizee besteht in der normativen Rechtfertigung der Welt (Immanenz) angesichts anderer Möglichkeiten, wie diese sein könnte und – aus einer kritischen Perspektive – auch sein sollte (Transzendenz).

Neben dem Zusammenhang zwischen dem rationalistischen Modell von Krisenlösung und der Einsicht, dass in unserem Fall für konspiratives Denken die Geheimnishaftigkeit wichtiger ist als das Entlarven, konnten durch die Interviewanalysen drei Themen identifiziert werden, die dem Verschwörungsdenken durchgängig als Grundlage dienen: Erstens werden die offiziellen Informationen über das Virus in Zweifel gezogen und eine vermeintliche massenmedial verzerrte Darstellung wissenschaftlicher Tatsachen kritisiert. Zweitens werden die politisch-administrativen Maßnahmen als absichtliche *„Angstmacherei“ oder „Panikmache“* bezeichnet, um sie als unnötige Hysterie zu kritisieren. Und drittens wird diese unterstellte systematische Erzeugung von Angst und Panik als Weg in die totale Überwachung und ein diktatorisches Kontrollregime kritisiert. Allen drei Kritiken entspricht umgekehrt eine spezifische Selbstdarstellung: Erstens sehen sich die Kritiker:innen als jemand, der sich nicht täuschen lässt und stattdessen selbst recherchiert; zweitens als jemand, der sich nicht einschüchtern lässt und sich dem heroischen Widerstandskampf verpflichtet fühlt;^19^ und drittens als jemand, der sich nichts vorschreiben lässt und sich resolut für Selbstbestimmung und Eigenverantwortung einsetzt.

b. Esoterik und Anthroposophie

Die zweite Komponente der *conspirituality* ist neben dem Verschwörungsdenken die Esoterik als alternative Form von Spiritualität. Auf die umfangreiche und intensive religionssoziologische und kulturhistorische Debatte zu diesem schwierigen Begriff kann hier nicht näher eingegangen werden.^20^ Für unsere Zwecke genügt als knappe Arbeitsdefinition von Esoterik: „claims of higher knowledge and ways of accessing this knowledge“ (von Stuckrad 2005, S. 88). Das „Höhere“ dieses Wissens bezieht sich auf den Anspruch, eine transzendentale Realität zu erkennen, was nur spezifisch Eingeweihten möglich ist. Wichtiger ist hier aber, dass es sich bei Esoterik um ein Wissen handelt, das sich selbst als „stigmatized knowledge (forgotten, superseded, ignored, rejected, suppressed)“ (Ward und Voas 2011, S. 116) sieht, sich gar als verbotenes, jedenfalls als irgendwie oppositionelles Wissen versteht (vgl. Asprem und Dyrendahl 2015, S. 371). Wie erwähnt, ist auch die Maßnahmenkritik nicht einfach eine Kritik an einer bestimmten Sache, sondern eine Kritik, die sich selbst als Kritik versteht und auch unbedingt so verstanden werden will. Esoterik begreift sich selbst als Gegen-Wissen, als alternatives Sonderwissen, als alternative Spiritualität, eingebettet in ein alternatives Weltbild. Die Alternativität als solche ist es, was zählt – ähnlich wie es auf den konkreten Inhalt der Kritik weniger ankommt als auf die Tatsache, dass es sich eben um Kritik handelt.

Im vorliegenden Beitrag wird auch die Anthroposophie als Esoterik verstanden, da sie ihre Vorstellung von alternativer Spiritualität teilt, die sich von der traditionellen Kirchenreligion absetzt, den Bezug zu einer transzendenten Wirklichkeit aber nachdrücklich betont. Im Kontext unserer Studie von besonderer Bedeutung ist dabei eine auffällige Hochschätzung des Natürlichen, wie z. B. des natürlichen Immunsystems. So etwa die Erziehungswissenschaftsstudentin: *„Aber, wenn es wirklich um Gesundheit geht, dann kann ich nicht verstehen, warum nicht gesagt wird, hey liebe Leute, es geht um Gesundheit, bitte macht viel Sport oder bitte bewegt euch, bitte geht jeden Tag an die frische Luft, trinkt genug Wasser, esst (..) genug Obst und Gemüse, von mir aus noch supplementiert Vitamin D3. Das wird nicht gesagt und da frag’ ich mich, geht es wirklich um Gesundheit? Geht es wirklich um die Stärkung des Immunsystems, weil das gehört nämlich dazu. Eine gesunde, ausgewogene Ernährung, Bewegung, frische Luft, Sonnenlicht etcetera pp.“ *Ein anderes Beispiel für esoterische bzw. anthroposophisch inspirierte Denkmuster findet sich im Interview mit der Lehrerin, die mehrmals betont, dass es die Angst vor dem Virus sei, die krankmache, nicht das Virus selbst. So sage sie ihren Schüler:innen: *„Man darf keine Angst haben, es braucht keine Angst (…) sondern wir schauen, wir beobachten lieber und schauen, was da passiert und dann kann man das besser einordnen, aber keine Angst, weil Angst, habe ich dann so gesagt, Angst macht krank. Und das haben sie sich gemerkt tatsächlich, also das haben sie bei jeder Gelegenheit wiederholt bei den Eltern“*.

Für die Analyse der esoterischen Vorstellungen ist es zentral, dass dabei einerseits die Wichtigkeit des Intuitiven und Gefühlvollen hervorgehoben wird, dass es sich aber andererseits nicht einfach um eine irrationalistische Position handelt, sondern um eine, die sich ihrerseits rationalistisch präsentiert. So verstand etwa Rudolf Steiner seine Lehre explizit als wissenschaftlich „exakte Clairvoyance“ (Zander 2019: 829). Allgemein ist es der Esoterik wichtig, dass es wissenschaftliche Beweise dafür gebe, dass entsprechende Praktiken einen (mehr oder weniger eindeutig) messbaren positiven Effekt auf das Wohlbefinden auszuüben vermögen.^21^ Idealtypisch findet sich diese Haltung in einem Interview mit der Erziehungswissenschaftsstudentin, die sich allgemein als spirituell, empathisch und intuitiv bezeichnet und spezifisch auf die Corona-Maßnahmen bezogen *als „sehr kritisch“*. Paradigmatisch lässt sich am folgenden Zitat ihr Bezug auf Rationalismus und Affektivität zeigen: *„Ich gucke immer, wo sind die Quellen einmal her, das auf jeden Fall. Dann guck ich auch, was fühlt sich für mich stimmig an“*. Auf der einen Seite legt sie damit eine wissenschaftliche Haltung an den Tag, indem sie Quellen prüft, auf der anderen Seite verlässt sie sich zur Beurteilung letztlich auf ihre Gefühle. Ähnlich auch die Psychologin: *„Und ich habe mir halt so viele Studien eben auch zu Masken durchgelesen und ein bisschen ist es aber auch so ein Bauch-Gefühl oder so ’ne Mischung aus Bauch und normaler Menschenverstand, würde ich mal sagen.“*^22^

c. Zusammenhang von Verschwörungsdenken und Esoterik

Vor dem Hintergrund der dargestellten Ergebnisse ergibt sich die Frage, was die Einheit von Verschwörungsdenken und Esoterik ausmacht, was es also ermöglicht, sie zur *conspirituality* zu verbinden. Schließler et al. (2020) arbeiten dafür mit psychoanalytischen Begriffen. Sie gehen davon aus, dass es sich bei der *conspirituality* um ein Narrativ handle, „das Angst, Überforderung und Ohnmacht bindet und dem (regressiven) Wunsch Raum gibt, in einer als kontrollierbar empfundenen Welt oder einem versöhnten (Ur‑)Zustand mit der Natur zu leben“. Sie erwähnen außerdem die „Kompensation von narzisstischen Kränkungen“ und die „Möglichkeit zur narzisstischen Befriedigung“ (Schließler et al. 2020, S. 295). Diese Perspektive kann vor dem Hintergrund der hier dargestellten Ergebnisse durch eine kultursoziologische Deutung ergänzt werden. Eine zentrale Gemeinsamkeit von Verschwörungsdenken und Esoterik stellt das Interesse am Geheimnisvollen dar: entweder an geheimen Mächten, die im Hintergrund die Welt beherrschen, oder an kosmischen Energien und anderen spirituellen Phänomene, deren Verständnis das okkulte Wissen von Eingeweihten bzw. „Erwachten“ voraussetzt. Die enge Verbindung von Verschwörungsdenken und Esoterik zur *conspirituality* wird in dieser Perspektive nicht durch individualpsychologische Kompensationsfunktionen erklärt, sondern durch den Bezug auf gesellschaftliche Rationalisierungsprozesse. Laut Weber beruhen diese auf der Überzeugung, „dass es also prinzipiell keine geheimnisvollen unberechenbaren Mächte gebe, die da hineinspielen, dass man vielmehr alle Dinge – im Prinzip – durch Berechnen beherrschen könne“ (Weber 2002, S. 488). Sowohl Esoterik als auch Verschwörungsdenken können als Versuche einer Wieder-Verzauberung durch Mystifikation gesehen werden. Ihre gemeinsame zentrale Aussage lautet aus dieser Sicht: Es gibt Geheimnisse bzw. Geheimnisvolles. Dass das Entlarven ein wichtiges Motiv des Verschwörungsdenkens ist, soll damit nicht bestritten werden.^23^ Im empirischen Material unserer Studie steht der Aspekt der Mystifikation allerdings deutlich im Vordergrund.

Der enge Zusammenhang zwischen dem rationalistischen Modell der Krisenlösung und verschwörungstheoretischen bzw. esoterischen Deutungsmustern, die durchaus irrationale Tendenzen aufweisen, erscheint zunächst widersprüchlich. Jedoch ist festzuhalten, dass es verkürzt wäre, Verschwörungsdenken und Esoterik als schlechterdings irrational zu verstehen. Verschwörungstheoretische Ansichten verweisen häufig auf den Anspruch, in investigativen und aufwendigen Recherchen zu gründen, und beruhen auf der präzisen Kenntnis von Fakten, die für wahrer gehalten werden als die offiziell kommunizierten. Zumindest in der Selbstbeschreibung geben sie sich selbst einen rationalistischen Anstrich. Das gilt ebenso für die Esoterik, der es wichtig ist, dass die Tatsache, dass mit wissenschaftlich-evidenzbasierten Mitteln nicht alles erklärt werden kann, wissenschaftlich bewiesen sei. Andererseits wäre es auch falsch, Verschwörungsdenken und Esoterik einfach als logische Entsprechung des rationalistischen Modells der Krisenlösung zu sehen. Die Verbindung ist durchaus widersprüchlich. In diesem Beitrag kann lediglich beschrieben werden, dass sie empirisch existiert. Eine schlüssige Erklärung dafür ist noch nicht gefunden, soll hier aber doch tentativ skizziert werden: Das rationalistische Modell der Krisenlösung dient dem Irrationalismus von Verschwörungsdenken und Esoterik zumindest rhetorisch als Steigbügelhalter. Das lässt sich durch die Umkehrung besser erkennen: Würden Inkonsistenz, Diskursrestriktionen und Fragen kalkulatorischer Verhältnismässigkeit nicht als Probleme gesehen werden, sondern als praktische Notwendigkeiten und als gewöhnliche Charakteristika des Umgangs mit ungewöhnlichen Situationen, könnten sie auch nicht als Indizien für die Annahme verwendet werden, dass etwas grundsätzlich nicht stimme bzw. dass es Dinge zwischen Himmel und Erde gebe, die der Verstand nicht erfassen kann. Die Tatsache etwa, dass Wissenschaft nicht alles erklären kann, ist aus Sicht der Wissenschaft vollkommen unstrittig. Sie wird als solche überhaupt interessant nur vor dem Hintergrund der Vorstellung, dass sie alles rational erklären können müsste. Dass Regierungshandeln nicht nach der formalen Logik der Widerspruchsfreiheit funktioniert, ist überhaupt informativ nur vor dem Hintergrund der Überzeugung, dass sie das eigentlich tun müsste. Dann kann es auch als Indiz gelesen werden, dass etwas anderes dahintersteckt bzw. dafür, dass die offizielle Kommunikation die wahren Absichten verschleiere.

### Stil: Formale Pathetik

Was den Stil der Maßnahmenkritik angeht, fällt ihre Pathetik auf, die eigentümlich abstrakt bleibt. Auf eine Formel gebracht: Wenig Substanzielles wird gesagt, das dafür aber leidenschaftlich. Der Begriff der Pathetik wird hier wertfrei verstanden als jede Art von Kommunikation mit der Funktion der Erregung möglichst vehementer Affekte bei den Hörer:innen, technisch ausgedrückt: als rhetorische Produktion von Passion. Besonders gut eignet sich dafür das Skizzieren von Untergangsszenarien (Ende von Demokratie und Rechtstaat) und das Ziehen möglichst spektakulärer Vergleiche.^24^ So wird in den Interviews wiederholt auf den Nationalsozialismus referiert, an einer Stelle behauptet die Lehrerin gar: *„Im Grund genommen sind wir da die Juden von damals oder?“* In diesem Sinne interpretiert es auch der 62-jährige Gebäudeverwalter: „*Und ganz persönlich bin ich der Meinung, dass das Tragen der Maske ein Unterwerfungsgeste sein kann: ‚Ich füge mich, weil es angeordnet ist‘. Es kann aber auch sozusagen der neue Hitlergruß sein: ‚Schau her, ich mache mit!‘“ *Auch Vergleiche mit dem totalitären Kommunismus werden angeführt, notorisch ist der Bezug auf China als Paradebeispiel für einen Überwachungsstaat mit *„total gläsernen und abhängigen Bürgern“*, so der 36-jährige Schreiner. Ein weiteres Beispiel aus dem Interview mit dem Ingenieur illustriert die rhetorische Radikalität treffend: *„Ich meine, ich will keine brasilianischen Verhältnisse, wo dann irgendwie, es dann irgendwelche Reichen-Ghettos gibt mit Stacheldrahtzaun und Bodyguards drum herum, und irgendwelche Slums, wo dann halt der Rest der Menschen leben. Ja, aber das wäre, das wäre dann das Ende, das logische Ende, dieser Entwicklung. Die wir jetzt, wenn wir nicht wirklich aufpassen, wo wir jetzt dahinsteuern“.* Und die 36-jährige Soziologin karikiert die Maskenpflicht folgendermaßen: *„Wenn ich Leute schon höre, ja, ist ja gar nicht so schlimm, die Masken, habe mich schon dran gewöhnt, finde ich einfach so, hey, hau ab! Dann geh in den Iran! Dort müssen sie auch die ganze Zeit Schleier anziehen, die Frauen. Es ist wirklich nichts mehr anders, ehrlich ges– einfach, dass dort (.) die Religion als Argument genommen wird und bei uns die Gesundheit, aber schlussendlich (…) ist’s mega ähnlich“.* In diesen Vergleichen manifestiert sich die Logik der formalen Pathetik deutlich. Formal ist sie, da über die gegenwärtige Situation nichts anderes gesagt wird, als dass sie vergleichbar ist mit anderen Zuständen und Ereignissen, die als absolut negativ gelten können. Pathetisch sind diese Vergleiche, da sie primär auf die Erzeugung affektiver Erregung ausgerichtet sind und weiter keinen informativen Gehalt vermitteln.

Neben dem Ziehen drastischer Vergleiche ist ein zweites Stilmittel der Maßnahmenkritiker:innen die Selbstinszenierung als heroische Widerstandskämpfer:innen, die standhaft ihre Meinung vertreten, keine Angst kennen und mutig bereit sind, dafür Opfer in Kauf zu nehmen.^25^ Dabei tritt ein libertäres Freiheitsverständnis zutage, so etwa im Interview mit dem 39-jährigen Wirtschaftsinformatiker: *„Mir muss niemand sagen, wann ich eine Maske zu tragen habe“*. In logischer Konsequenz bedeutet dies aber auch, dass von anderen kein Schutz erwartet wird: *„Kein Mensch muss wegen mir eine Maske tragen, um mich zu schützen, ich schütz mich selber.“ *Durch den Bezug auf heroischen Widerstand wird in dieser Sicht auch die Glaubwürdigkeit der Informationsquellen garantiert. Bewundert wird dabei vor allem die Opferbereitschaft. So etwa der Logistiker: *„Ja also mit outen meine ich einfach, dass man sich, dass man den Mut hat, also den Mut fasst und sagt: Okay ist gut ich mache jetzt eben ein Video, obwohl ich vielleicht damit meinen Job riskiere, weil ich mich da ein bisschen kritisch äußere.“* Romantisiert wird die bedingungslose Hingabe an ein Ideal, „no matter what it was“ (Berlin 1999, S. 9). Zur formalen Pathetik des Heroismus lässt sich auch die wiederholt analysierte Betonung zählen, dass die kritische Haltung schon von *„ganz am Anfang“* bestanden habe. Noch einmal die Rentnerin: *„Bin von Anfang an, ich bin aber schon immer mit allem so, dass ich immer zuerst mal etwas hinterfrage also ich bin nicht die, die etwas sieht und dann sagt: Woaaah boaaah das muss alles wahr sein. Sondern ich schaue immer grad, ich ziehe den Vorhang und schaue dahinter.“* Damit wird die eigene Unerschütterlichkeit und Standfestigkeit hervorgehoben und auch eine Differenz gezogen zwischen den wahrhaft kritischen Menschen und solchen, die nun vielleicht auf den Zug noch aufgesprungen sind, weil es unterdessen auch legitim (wenn nicht gar: populär) geworden ist, zumindest ein bisschen dagegen zu sein.

Eine dritte Variante formaler Pathetik ist der Bezug auf Kinder, der uns in seiner Regelmäßigkeit durchaus überrascht hat.^26^ So führt der Ingenieur aus: „*Was mit den Kindern gemacht wird, wenn denen jetzt Angst gemacht wird: Ja wenn du keine Maske anziehst, stirbt deine Oma, ne. Das ist ja also sorry. Ich meine, das ist ist ’ne Vergewaltigung von Kinderseelen“*.^27^ Auf einem Transparent an der Konstanzer-Demo war zu lesen: „Maskenpflicht ist Kindsmisshandlung“ (vgl. Frei et al. 2021). Diese Terminologie ist an Drastik kaum zu überbieten. Auch hier ist der Informationswert der Aussage äußerst gering, ihre Funktion ist einzig die Provokation affektiver Reaktionen. Kindesmissbrauch ist ein emotional so stark beladenes Thema, dass ein solcher Vorwurf auf Aufmerksamkeit hoffen kann – wenn er auch abgelehnt wird. Es kann darauf fast nicht nicht reagiert werden.

## Gesellschaftstheoretische Einbettung

Nachdem der Inhalt und der Stil der Maßnahmenkritik beschrieben sind, stellt sich die weitergehende soziologische Frage, was diese Kritik über die moderne Gesellschaft aussagt. Eine Möglichkeit, sie zu beantworten, soll abschließend skizziert werden. Der Ausgangspunkt ist dabei Eisenstadts Weiterentwicklung der Weberschen Rationalisierungstheorie. Eisenstadt bestimmt den Prozess der Rationalisierung als fundamentalen „Verlust der Grundlagen aller Gewissheit“ (Eisenstadt 2006, S. 38). Die zentrale Folge davon ist eine massive Amplifikation von Kritik und, kehrseitig dazu, die Erhöhung der Anforderungen an Legitimation gesellschaftlicher Herrschafts- und Machtbeziehungen. Nicht nur politische Herrschafts- und Machtstrukturen können sich nicht mehr auf traditionale Selbstverständlichkeiten berufen. Zumindest prinzipiell ist in der modernen Gesellschaft jede Institution hinterfragbar geworden: Religion, Wissenschaft, Recht, Ökonomie, Erziehung, Kunst, Liebe etc. Und erst die Moderne hat auch die Möglichkeit der Kritik an der gesellschaftlichen Ordnung *als Ganze* hervorgebracht. Damit ist ihr die charakteristische Widersprüchlichkeit eingeschrieben, sich gerade dadurch zu legitimieren, dass sie Kritik ermöglicht, die sich fundamental gegen ihre eigenen Legitimitätsansprüche richten kann.

In den aktuellen Protesten gegen die Corona-Maßnahmen zeigt sich nun eine bemerkenswerte Zuspitzung der von Eisenstadt beschriebenen Entwicklungstendenz. Auch ein fundamentaler Verlust gesellschaftlicher „Gewissheitsgrundlagen“ führte in der Vergangenheit nicht dazu, dass Kritik auf jegliche Form von Gewissheit verzichtet hat. Was Wittgenstein über den Zweifel sagt, gilt auch für Kritik: „Unsre Zweifel beruhen darauf, dass gewisse Sätze vom Zweifel ausgenommen sind“ (1990, S. 186). „Ein Zweifel, der an allem zweifelte, wäre kein Zweifel“ (Wittgenstein 1990, S. 209) – sondern Verzweiflung (vgl. Oevermann 2016, S. 90). Im folgenden Zitat aus dem Interview mit dem Ingenieur zeigt sich diese Verzweiflung deutlich: „*Ich hab so ’ne Art so ’ne Mischung zwischen Wut und Entschlossenheit im Bauch, aber manchmal ist es auch Resignation, ich weiß es nicht.“* In den Corona-Protesten manifestiert sich eine Kritik, die keine andere Gewissheit mehr kennt als die Kritik selbst.^28^ So etwa der Schreiner: *„Ich mein, ich find da diesen Querdenkengrundsatz ganz gut. Glaube wenig, denke selbst, hinterfrage alles. Das finde ich gar nicht so schlecht. Also wirklich 100* *% Vertrauen hab’ ich jetzt in keine Medien, also alle Informationen nehme ich erst mal an, aber immer unter Vorbehalt.“* Auch die Erziehungswissenschaftlerin vertraut überhaupt keinen Quellen mehr, sondern nur noch sich und engen Bezugspersonen: *„Ich glaube mittlerweile echt nur noch Sachen, die ich mit meinen eigenen Augen gesehen hab’ oder die Vertraute von mir, mein Partner, meine Eltern gesehen haben.“ *Die Kritik an den Maßnahmen der politischen Entscheidungsträger funktioniert als Vorwand eines „Generalverdachts“ (Boltanski 2010, S. 169) an der offiziellen Darstellung der gesamten gesellschaftlichen Wirklichkeit.^29^ Gewiss ist nur noch, dass nichts mehr gewiss ist. Die daraus abgeleitete Pflicht, ständig alles zu hinterfragen, verweist auf ein grundlegendes Glaubwürdigkeitsproblem der modernen Gesellschaft.^30^ Wenn indessen alles hinterfragt wird, insbesondere die sogenannten Mainstream-Medien, dann stellt sich die Frage, wodurch die eigenen Quellen Glaubwürdigkeit beanspruchen können. Entweder vertrauen die Befragten nur der eigenen Expertise oder erachten andere kritische Stimmen genau deshalb als glaubwürdig, weil sie aus ihrer Perspektive eben kritisch sind. Als Beispiel dafür ein prägnanter Auszug aus dem Interview mit dem Logistiker:

I1: Und an welchen Kriterien kannst du unterscheiden welche Quellen vertrauensvoll sind und welche nicht, also weil auf Youtube oder insgesamt werden ja verschiedene, ja verschiedene Darstellungen sozusagen-

B: Genau das ist eine gute Frag. Also ich (.) sag jetzt mal bin offener für ein wenig kritischere Stimmen. (..) /I2: Was Was-/ja kritische Stimmen

I2: Und was macht- was macht für dich eine kritische Stimme aus?

B: Ja jemand, der sich jetzt öffentlich auch das Ganze in Frage stellt (4.0) /I2: Mhm/(.) und auch, ja, die sich sozusagen wie- wie outen die- die auch öffentlich über, sei es über Youtube oder über eine Demo (.) genau das kritisiert. Ja

Hier zeigt sich exemplarisch, dass die Glaubwürdigkeit *der* Kritik einer Glaubwürdigkeit *durch* Kritik entspricht.^31^ Die Maßnahmenkritiker:innen haben zwar ihre eigenen Gewissheiten, was sich insbesondere in der weitgehend kritiklosen Akzeptanz der einschlägigen Quellen ausdrückt, die als unhinterfragbar dargestellt werden. Bemerkenswert ist zudem das Selbstbewusstsein, mit dem die Akteure als Expert:innen auftreten, wenn sie Erkenntnisse hinterfragen, die in der wissenschaftlichen Fachwelt auf Konsens zählen können. Was mit dem Bezug auf Eisenstadt hier gesagt werden soll, ist also nicht, dass es für die Maßnahmenkritiker:innen keine Gewissheiten mehr gibt, sondern dass diese Gewissheiten darauf beruhen, dass nichts mehr gewiss ist. Anders gesagt: Die Glaubwürdigkeit der eigenen Quellen beruht darauf, dass sie die Glaubwürdigkeit der offiziellen Darstellungen bestreiten. Entsprechend bezieht die Kritik ihre Legitimation primär daraus, *dass* sie eben Kritik ist.

Die Ergebnisse der empirischen Analysen zeigen, dass die Kritik an den Corona-Maßnahmen von der Vorstellung ausgeht, dass es perfekt rationale Lösungen der Krise geben könne. Die Tatsache, dass dies nicht der Fall ist, wird dann als Indiz dafür genommen, dass etwas nicht stimmen könne. Auf diese Weise können die Maßnahmen als Problem konstruiert werden, auf das die Kritik mit einer Haltung reagiert, die als *conspirituality* bezeichnet werden kann, als Kombination verschwörungstheoretischer und esoterischer Motive. Beide Denkmuster sind charakterisiert durch ihr Interesse am Geheimnisvollen, was sich wiederum als Reaktion auf die Dynamik der Entzauberung verstehen lässt. Der Stil der Maßnahmenkritik kann als formale Pathetik bestimmt werden: Wenig wird gesagt, das dafür aber leidenschaftlich. Gleichzeitig dient diese formale Pathetik zur Vergemeinschaftung der Protestbewegung (Frei et al. 2021). Aus ihrer eigenen Perspektive sind die Maßnahmenkritiker:innen die wahren Retter:innen von Freiheit und Demokratie, weil sie erkannt haben wollen, dass etwas anderes dahinterstecke, und sie mutig ihren Widerstand auf die Straße tragen. Unser empiriebasierter Vorschlag für die gesellschaftstheoretische Einbettung des untersuchten Gegenstands bestimmt die Maßnahmenkritik als Ausdruck eines übergreifenden Prozesses der Erosion aller Gewissheiten. Letztlich bleibt der Kritik als Grundlage nichts mehr übrig als der Eigenwert der Kritik als solcher.

## Footnotes

1 Das zeigen zumindest die Zahlen für Deutschland. In der Schweiz verhält es sich insofern anders, als die nationalkonservative SVP von Anfang an die stärkste Partei bei den Maßnahmenkritiker:innen gewesen ist (Nachtwey et al. 2020).

2 Die Schwierigkeiten der politischen Verortung der Corona-Proteste zeigt auch Hanloser (2021) am Beispiel der Zeitung *Demokratischer Widerstand*. Nach Buchmayr (2019, S. 379) ist das ein allgemeines Merkmal verschwörungstheoretischer Gruppen: „Begriffe wie links, rechts, progressiv und konservativ spielen für das Selbstverständnis der Verschwörungstheoretikerinnen keine Rolle. Ihre gemeinsame Identität gewinnen sie ausschließlich aus der Ablehnung der Orthodoxie bzw. des ‚Mainstreams‘“.

3 Zitate aus den Interviews werden in Anführungszeichen und kursiv gesetzt. Alle Zitate bleiben nahe am Wortlaut, sind zugunsten besserer Lesbarkeit sprachlich aber leicht geglättet: Verzögerungslaute sind gestrichen, Satzzeichen wurden eingefügt, Satzabbrüche, Stottern etc. größtenteils gestrichen. Die schweizerdeutschen Interviews wurden schriftdeutsch transkribiert und in dieser Form analysiert.

4 Vgl. auch Hentschel (2021, S. 69f.).

5 In diesem Sinne kann man von einer „normativen Unordnung“ (Nachtwey 2016) sprechen, Teune (2021) sieht eine Ähnlichkeit mit den „Montagsmahnwachen“.

6 Dass diese Kombination keineswegs so überraschend ist, wie es Ward und Voas darstellen, sondern esoterische und konspirationistische Denkmuster eine unauflösbare, historisch gewachsene Verflechtung darstellen, zeigen überzeugend Asprem und Dyrendahl (2015).

7 Aufgrund einer fehlenden Einverständniserklärung konnte ein Interview nicht verwendet werden, weshalb in der Übersicht nur 15 aufgeführt werden. Ein Großteil der Interviews wurden zwischen Oktober 2020 und Januar 2021 geführt. Wir danken allen Teilnehmer:innen der Arbeitsgruppe „Coronaproteste und Verschwörungsdenken“, die im Herbstsemester 2021 im Fachbereich Soziologie unter der Leitung von Dr. Nadine Frei durchgeführt wurde.

8 Mit Bourdieu ließe sich das als Konfrontation der praktischen Logik mit den Anforderungen der theoretischen Logik bestimmen (1993, S. 157).

9 Nicht nur zeigt sich hier die rationalistische Vorstellung, dass es zwischen politischer Praxis und wissenschaftlicher Forschung keine Widersprüche geben solle, sondern auch innerhalb der Wissenschaft wird Widersprüchlichkeit als etwas Problematisches angesehen. Allerdings sind Widerspruch, Kritik und das Scheitern von Hypothesen konstitutiver Bestandteil wissenschaftlichen Fortschritts.

10 Im Schweizerdeutschen erfüllt das einem Satz nachgestellte „oder?“ die Funktion einer rhetorischen Nachfrage, ob das Gegenüber verstanden hat, vergleichbar mit dem deutschen „ne?“. Es zielt auch auf das Erheischen von Zustimmung.

11 Während in den Analysen (a) durchweg rekonstruiert werden konnte, gilt das für (b) nicht.

12 „Der Glaube an Verschwörungserzählungen füllt allerdings keine Lücke in ethischer Hinsicht, sondern versucht – mit einer Art ‚Hyper-Rationalität‘ –, der Wahrheit mit einer übertriebenen Bereitschaft für logisches Denken, falsche Deduktionen und dem Duktus positivistischer Wissenschaft und Faktenbesessenheit auf die Spur zu kommen“ (Schließler et al. 2020, S. 294).

13 Butter (2020). Amlinger und Nachtwey „interpretieren die Zunahme konspirativer Denkmuster im Zuge der Corona-Pandemie in diesem Sinn als eine versuchte epistemische Resouveränisierung, die die Kränkungen des spätmodernen Subjekts kitten soll“ (2021).

14 Umfangreiches Material zu Verschwörungstheorien sowie die Explikation einer spezifisch wissenssoziologischen Theorie findet sich in Anton et al. (2014).

15 Eine Eindeutigkeit, ob es im Zuge der Corona-Krise zu einer Konjunktur des Verschwörungsdenkens gekommen ist, besteht nicht: Nach Roose (2020) hat die gesellschaftliche Verbreitung von Verschwörungstheorien durch die Corona-Krise nicht zugenommen, sondern sogar leicht abgenommen und sinkt vom erstaunlich hohen Wert von 30 auf 24 %. Decker et al. (2020, S. 201) konstatieren 2020 eine Zunahme der Verschwörungsmentalität im Vergleich mit der Befragung im Jahr 2018. So würden 38,4 % der Befragten eine manifeste Verschwörungsmentalität aufweisen. Da Verschwörungsmentalität und Verschwörungstheorie aber nicht das Gleiche sind, sind diese Befunde nicht direkt vergleichbar. Für unsere Zwecke ist die quantitative Verbreitung indessen eine nachrangige Frage.

16 So auch Lamberty und Imhoff: „Es geht also weniger um die Behauptung einer konkreten Verschwörung, sondern um den Ausdruck eines Weltbilds, in dem einige wenige sich zu ihrem Vorteil und dem Nachteil der Allgemeinheit verschworen haben – wie genau ist oft nebensächlich“ (2021, S. 203).

17 Der Ingenieur im Zusammenhang mit 9/11: *„Ich weiß nicht, wie’s passiert ist, aber ich weiß nur, dass es so wie’s erzählt wird, nicht physikalisch einfach nicht sein kann.“ *Vgl. zu diesem Aspekt von Verschwörungstheorien auch Kuhn: „Nur wer weiß, dass er nicht weiß, kann dies zum Anlass für Verdacht nehmen“ (2010, S. 121).

18 Geschichte wird so als plan- und kontrollierbar betrachtet, Kontingenz und Zufall werden dadurch eliminiert (Butter 2020, S. 28). Siehe zu diesen „existenzielle[n] Motive[n]“ auch Lamberty und Imhoff (2021, S. 204).

19 Vgl. Hentschel (2021, S. 81f.).

20 Einen tieferen Einblick in die Thematik ermöglicht der Sammelband von Neugebauer-Wölk et al. (2013).

21 Das gilt auch schon für die Theosophie, aus der dann Steiners Anthroposophie hervorging: „Das vom Spiritismus übernommene Feindbild, der ‚Materialismus‘, blieb allen Theosophen in den Blick gebrannt. Als Kind des 19. Jahrhunderts trat die Theosophie an, diesen Feind mit seinen eigenen Waffen zu schlagen, und als dessen schärfste galt der Wissenschaftsanspruch“ (Zander 2001: 433).

22 Zu diesem Ergebnis kommen auch Pantenburg et al. „Erfahrungswissen, intuitives und gefühltes Wissen sowie deren Funktionen und die Mechanismen ihrer Bewährung sind im Kontext dieser ‚Querdenken‘-Proteste von besonderer Relevanz“ (2021, S. 3).

23 Vgl. z. B. Roose: „Verschwörungstheorien beschreiben und kritisieren nicht allein Machtunterschiede, sondern sie beanspruchen, eine geheime, niederträchtige Verschwörung zu entlarven“ (2020, S. 7).

24 Hentschel (2021, S. 66) spricht in diesem Kontext von *affective drives*: „dramatische Temporalitäten. Immer steht etwas Wichtiges auf dem Spiel, das verloren werden oder kaputt gehen kann: unsere Demokratie oder die Zukunft unserer Kinder. Und eine furchterregende Realität wartet auch schon: die Diktatur oder die Sklaverei“.

25 Vgl. zur Opferbereitschaft als zentrales Element des Heroischen Bröckling (2015, S. 14).

26 Vgl. Hentschel (2021, S. 65).

27 Aus dem Interview mit der Rentnerin:* „Dort hat’s mir einfach voll den Nuggi rausgehauen *(schweizerdeutsche Redewendung mit der Bedeutung: da bin ich ausgerastet, Anm. die Autor:innen)* oder, also Kinder, die die jetzt in Deutschland die Masken tragen müssen, die haben zum Teil – die Säuglinge tragen Masken, da drehe ich durch*“. Einen ähnlichen Kipppunkt markiert die 46-jährige Psychologin: *„Also irgendwann fing das dann ja an mit den Masken und Maskenpflicht, auch an der Schule. Und das war der Punkt wo ich echt (..), da war’s bei mir echt komplett überschritten so. Weil meine Tochter, die ist halt acht, und mein Sohn ist zwölf und ich habe wirklich viel gelesen. Ich habe mir auch, ich weiß nicht, 50 ungefähr, so Studien zu Masken angeguckt. […] Und für Kinder gibt es, oder gibt es ja gar keine richtige Studie und so. Und da war ich halt extrem angenervt so erst und dann irgendwann bin ich auch richtig wütend geworden und auch richtig – und da hatte ich so einen totalen Schub.“* Von unserer Studie (Nachtwey et al. 2020) hat sie über den Telegram-Kanal „Eltern stehen auf“ erfahren.

28 Vgl. Holzer (2021, S. 19): „Der Zweifel bleibt zurück, selbst wenn er außer auf die Begrenztheit allen Wissens kaum auf Gründe verweisen kann“.

29 Vgl. Harambam und Aupers (2017, S. 6–7).

30 Buchmayr (2019, S. 382): „Man müsse stets kritisch an sich arbeiten, sich hinterfragen und alles, was man zu wissen glaube, anzweifeln.“

31 Vgl. Holzer (2021, S. 20).
